# Late Loeys-Dietz Syndrome Diagnosis in an Adolescent With Severe Phenotype

**DOI:** 10.1016/j.jaccas.2025.105357

**Published:** 2025-09-10

**Authors:** Lorraine James, Nazia Husain, Sarah Jurgensmeyer Langas, Joshua Baker, Kristen Nelson McMillan, Luca Vricella, Joseph Camarda

**Affiliations:** a Ann & Robert H. Lurie Children's Hospital, Chicago, Illinois, USA; b Northwestern Feinberg School of Medicine, Chicago, Illinois, USA; c Advocate Children's Hospital, Chicago, Illinois, USA

**Keywords:** aorta, cardiac magnetic resonance, cardiovascular disease, chronic heart failure, computed tomography, dissection, echocardiography, genetic disorders, insufficiency, valve repair

## Abstract

**Background:**

Loeys-Dietz syndrome (LDS) is a rare connective tissue disorder (CTD) with musculoskeletal, craniofacial, and cardiovascular features with a prevalence of approximately 1:50,000. Morbidity and mortality often occur earlier in patients with LDS compared to patients with other CTDs.

**Case Summary:**

We present a teenager with subacute heart failure, 4/6 holosystolic murmur with diastolic rumble, facial differences, and arachnodactyly. She had genotype-positive, phenotype-positive LDS including an atrial septal defect, severely dilated great arteries/ventricles, and depressed systolic function, requiring prompt medical and surgical therapy.

**Discussion:**

To our knowledge, this is the first case of LDS with such dramatic atrial and ventricular dilation, likely the result of shunting, valve regurgitation, and underlying CTD.

**Take-Home Messages:**

LDS can be aggressive in young patients; screening and diagnosis should be prompt when LDS is suspected. Elective repair of the aorta is indicated at smaller absolute diameters in LDS. Intervention lowers risk of a sudden, potentially life-ending cardiac event.

## Graphical Abstract

**Figure:**
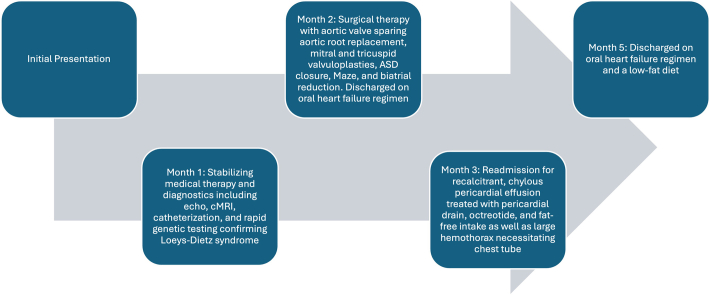

**Visual Summary:**
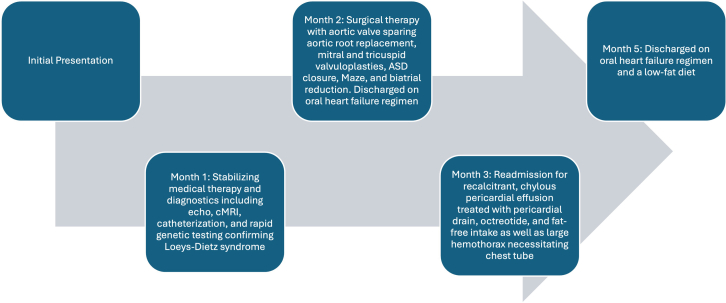
Case Timeline After presentation, priority was given to stabilizing the patient with medical therapy and pursuing diagnostic investigations. Surgical repair was done 32 days after presentation. Her postoperative course was complicated by both chylous effusion and hemothorax.

## History of Present Illness

A 13-year-old girl presented to the emergency department with 3 months of intermittent, left-sided, stabbing chest pain and exercise intolerance. There had been no cyanosis, syncope, respiratory distress, or weight loss. It was becoming harder for her to walk. Three weeks before presentation, she had immigrated to the United States from Mexico.

**Figure 1:**
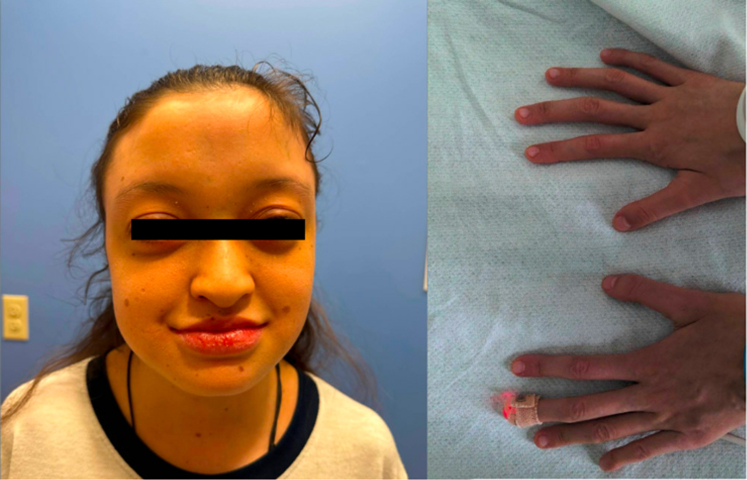
Physical Examination Findings The physical examination demonstrated distinct facial differences such as hypertelorism, flattened midface, wide nasal bridge, and thin philtrum as well as arachnodactyly, consistent with stigmata of a connective tissue disorder.

On initial examination, she had normal vital signs, including normal saturations, bounding peripheral pulses, a thrill with a 4/6 holosystolic murmur at the left upper sternal border, and a 2/4 diastolic rumble. She had visible cardiac contractions through a thin chest wall and jugular venous distention. Additionally, she had hypertelorism, flattened midface, wide nasal bridge, thin philtrum, low-set and posteriorly rotated ears, high arched palate with a bifid uvula, scoliosis, translucent skin, and arachnodactyly (Figure 1).

## Past Medical History

Per family report, a murmur was detected at birth, associated with a diagnosis that the parents could not recall. The patient attended follow-up with a pediatric cardiologist for a year beginning at age 7, when she was started on an oral heart failure regimen. Her family was told that she would require surgical cardiac intervention for her condition but that she would do poorly given her “small size.” The family was not able to have continued follow-up with the cardiologist owing to financial difficulty but continued the patient's medications that were prescribed originally (captopril, furosemide, and spironolactone).

## Differential Diagnosis

Her clinical presentation suggested subacute to chronic heart failure and a possible genetic syndrome. She was admitted to the cardiac intensive care unit for heart failure management; during this time, she developed ectopic atrial tachycardia, ventricular tachycardia, and atrial fibrillation. Even before investigations, an unspecified cardiomyopathy was a likely differential diagnosis given her slow progression of symptoms, arrhythmia, and lack of stigmata of chronic cyanotic heart disease.

## Investigations

**Figure 2:**
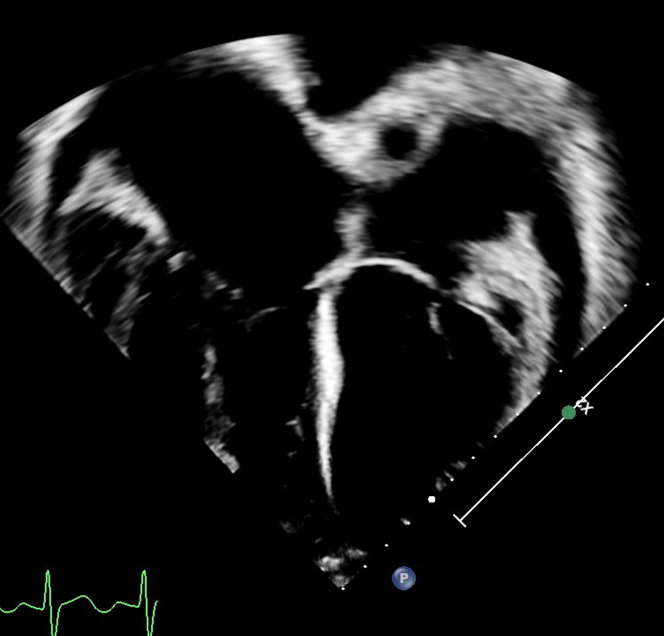
Echocardiogram Findings An apical 4-chamber image from her echocardiogram showed bilateral, gigantic atria with aneurysmal dilation lending the image its telltale cartoon heart shape.

Chest x-ray showed a massive cardiac silhouette with bilateral hilar enlargement. An echocardiogram showed bilateral gigantic atria with aneurysmal dilation (Figure 2), a large atrial septal defect (ASD) with bidirectional flow, severe pulmonary artery and aortic root dilation, severe ventricular dilation and dysfunction, and moderate pulmonary hypertension with moderate tricuspid and mitral regurgitation.

**Figure 3:**
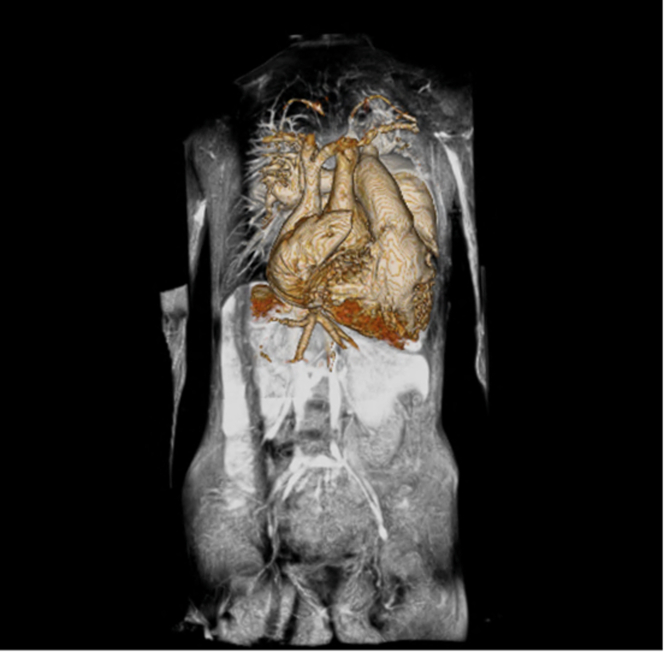
Initial Magnetic Resonance Imaging Findings Magnetic resonance imaging demonstrated the sheer amount of space within the thorax taken up by the patient's large heart and great vessels.

Cardiac magnetic resonance confirmed and quantified echocardiogram findings of a severely dilated aortic root and pulmonary arteries, severely dilated ventricles, severely depressed global systolic function, and small pericardial and pleural effusions (Figure 3). Specifically, her aortic root measured 56.4 × 56.2 × 56.6 mm, translating to a *z*-score of +15.9. There was tortuosity of the vertebral arteries; however, there was no focal stenosis or aneurysm of the head and neck vessels. The large secundum-type ASD was again noted, contributing to the extreme dilation of the cardiac chambers due to intracardiac shunting. Cardiac catheterization revealed normal systolic and diastolic function, low pulmonary vascular resistance (<3 indexed WU), a Q_p_/Q_s_ of 3.5:1 indicative of a substantial left-to-right shunt, and elevated mean pulmonary artery pressures (34-40 mm Hg) in all conditions of vasodilatory testing. The normal diastolic function effectively ruled out a primary cardiomyopathy.

Based on these data, a connective tissue disorder (CTD) was highly suspected, with specific concern for Loeys-Dietz syndrome (LDS). Given the acute presentation and anticipated impact to clinical care, rapid broad genetic testing was recommended, and her family was consented for trio genome sequencing. Testing identified a heterozygous pathogenic variant in *TGFBR2* (c.1135G>T, p.D379Y), diagnostic for LDS. The pathogenic *TGFBR2* variant was not identified in the genetic sample of either parent, suggesting it was a new variant in the patient. However, her parents reported their 7-year-old daughter had a similar phenotype, so targeted familial variant testing was completed, which identified the same variant. The presence of the variant in 2 full siblings with confirmed parentage and sample integrity was diagnostic of parental mosaicism.

## Management

**Figure 4:**
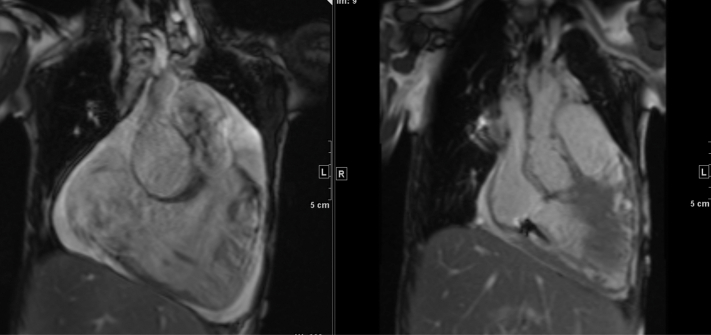
Effects of Valve-Sparing Root Replacement The valve-sparing root replacement changed the dimensions of the aortic root from severely dilated (56.4 × 56.2 × 56.6 mm, *z*-score: +15.9) to only mildly dilated (31 × 33 × 33 mm, *z*-score: +3.3).

Initial management consisted of diuretics and afterload reduction to decrease valvular leakage. The patient’s aforementioned arrhythmia burden prompted initiation of amiodarone and synchronized cardioversion to sinus rhythm. After catheterization revealed low pulmonary vascular resistance, she was considered a candidate for surgical intervention from a risk perspective. She underwent valve-sparing root replacement (Figure 4), mitral valve repair, tricuspid valve repair, ASD closure, Maze procedure for arrhythmia, and biatrial reduction. Postoperative course was complicated by persistent chylous pericardial effusion.

## Outcome and Follow-Up

In the outpatient setting, the patient was restricted from fluoroquinolones, heavy weightlifting, isometric exercises, and contact/competitive sports, which is standard for patients with severe CTD involvement of the aorta. Subacute bacterial endocarditis prophylaxis was recommended given her valve interventions. Her long-standing lymphopenia, anemia, and chronic thrombi were also monitored by various subspecialists. She is currently 1 year and 6 months from her surgery and is on a stable oral heart failure oral regimen, antiarrhythmics, and a PDE5 inhibitor. She has no cardiac symptoms, such as chest pain, shortness of breath, exercise intolerance, or syncope. She requires continued surveillance of her aorta and a left subclavian artery aneurysm that has since developed.

## Discussion

There are several reported cases of late diagnosis of LDS, including some occurring as late as the seventh decade of life.^1^^,^^2^ Cardiac involvement in these patients was limited to aortic complications and mild ventricular dilation. Our case represents the first noted with such dramatic atrial and ventricular dilation, likely the result of extra flow from the ASD, valve regurgitation, and the underlying CTD. In patients with LDS, morbidity and mortality tend to occur at an early age, with complications developing at relatively smaller aortic dimensions compared with other aortopathies. Thus, the dimensions prompting aortic root replacement in patients with LDS are smaller (4.0-4.2 cm) than in patients with a dilated aortic root without CTD (5.0-5.5 cm).^3^ Our patient, with a root measuring 5.6 cm in the largest diameter, exceeded indication for surgical intervention. Earlier diagnosis of patients with LDS allows for earlier screening for complications and earlier intervention before vascular dissection/rupture.

Our patient's surgery included not only aortic root replacement but also valve interventions. Long-standing, unrepaired atrioventricular valve dysfunction leads to irreversible myocardial dysfunction, underscoring another reason to detect this disease early. In this case, late presentation was the aggravating factor for the degree of cardiac enlargement and dysfunction.

Rapid genetic testing confirmed the diagnosis in this instance. Apart from its prompt turnaround allowing for quick intervention, this testing helps differentiate when there is phenotypic overlap for presenting features of a CTD, which is crucial for prognosis, risk stratification, and nuanced management. For our patient and her family, the results were suggestive of parental mosaicism, given identification of the same pathogenic *TGFBR2* (c.1135G>T, p.D379Y) variant in both our patient and her sister with negative parental blood samples. Mosaicism, or the presence of multiple cell lines with different genotypes in 1 individual, can limit variant detection depending on which cell line is assessed in any given sample. Though this phenomenon of mosaicism in the parent of a patient diagnosed with LDS has been described rarely in case reports, its true incidence is unknown.^4^^,^^5^ These complex results emphasize the importance of counseling from a certified genetic counselor for interpretation of results, discussion about recurrence risk, and coordination of cascade testing to identify other at-risk family members.

Surgical repair was performed 32 days after presentation, during which time her medical management was optimized, and additional testing was pursued to gauge operability. She is only a year and a half from her surgery, so time will tell whether her cardiac surgical outcomes will align with existing data. At 1 institution that reported nearly 20 years of pediatric experience with valve sparing aortic root replacements comprising 100 patients, 39% had LDS. Perioperative valve-sparing root replacement mortality was 2%. Of the total 100 patients, 6 required late reintervention for pseudoaneurysms, and 8 underwent additional aortic surgery.^6^ The average time to reoperation was approximately 7 years. As our patient also required multiple valve interventions and a Maze procedure to decrease arrhythmia burden, the level of operative complexity and risk were also significantly increased.

## Conclusions

We hope that the report of this teenage patient enhances awareness of the aggressive nature of LDS in young patients, leading to earlier screening and diagnosis when there is clinical suspicion. This can allow for elective repair if indicated, which inherently lowers the risk of a sudden, potentially life-ending cardiac event in pediatric patients with this disease.

## Funding Support and Author Disclosures

The authors have reported that they have no relationships relevant to the contents of this paper to disclose.

**Take-Home Messages**

• LDS can be aggressive in young patients; screening and diagnosis should be prompt when LDS is suspected.
• Elective repair of the aorta is indicated at smaller absolute diameters in patients with LDS. Intervention lowers risk of a sudden, potentially life-ending cardiac event.
